# Developing and evaluating multimedia information resources to improve engagement of children, adolescents, and their parents with trials (TRECA study): Study protocol for a series of linked randomised controlled trials

**DOI:** 10.1186/s13063-017-1962-z

**Published:** 2017-06-08

**Authors:** Jacqueline Martin-Kerry, Peter Bower, Bridget Young, Jonathan Graffy, Rebecca Sheridan, Ian Watt, Paul Baines, Catherine Stones, Jennifer Preston, Steven Higgins, Carrol Gamble, Peter Knapp

**Affiliations:** 1 Department of Health Sciences, University of York, Heslington, YO10 5DD York, UK; 2 MRC North West Hub for Trials Methodology Research, NIHR School for Primary Care Research, University of Manchester, M13 9PL Manchester, UK; 3 MRC North West Hub for Trials Methodology Research, Department of Psychological Sciences, Institute of Psychology, Health and Society, University of Liverpool, L69 3GB Liverpool, UK; 4 Department of Public Health and Primary Care, University of Cambridge, Institute of Public Health, Forvie Site, Robinson Way, CB2 0SR Cambridge, UK; 5 Department of Health Sciences and the Hull York Medical School, University of York, Heslington, YO10 5DD York, UK; 6 Paediatric Intensive Care Unit, Alder Hey Hospital, L12 2AP Liverpool, UK; 7 School of Design, Clothworkers’ Central, University of Leeds, LS2 9JT Leeds, UK; 8 NIHR Alder Hey Clinical Research Facility, University of Liverpool, Institute in the Park, Alder Hey Children’s NHS Foundation Trust, Eaton Rd, L12 2AP Liverpool, UK; 9 School of Education, University of Durham, DH1 3LE Durham, UK; 10 Institute of Translational Medicine, University of Liverpool, L69 3GB Liverpool, UK

**Keywords:** Multimedia, Intervention, Trial participation, Child, Adolescent, Information, Recruitment, Retention, Decision-making, Consent, Parent

## Abstract

**Background:**

Randomised controlled trials are widely established as the best method for testing health interventions whilst minimising bias. However, recruitment and subsequent retention of children and adolescents in healthcare trials is challenging. Participant information sheets are often lengthy and difficult to read and understand. Presenting key information using multimedia may help to overcome these limitations and better support young people and their parents in deciding whether to participate in a clinical trial.

**Methods:**

The TRECA (TRials Engagement in Children and Adolescents) study has two phases. The first phase involves a qualitative study with children and adolescents and their parents to inform the development of multimedia information resources and iterative user testing to refine the resources. The second phase will embed the use of the multimedia information resources into six host trials in the United Kingdom. Patients and parents approached to participate in the host trials will be randomly allocated to either use the multimedia information resource in conjunction with standard participant information sheets, the multimedia information resource alone, or the standard participant information sheets alone. The primary outcome will be the effect of the multimedia information resources on recruitment into trials. Other outcomes measured include the effect of multimedia information resources on retention of participants into the host trials and the impact on family members’ decision-making processes, when compared to standard participant information sheets alone.

**Discussion:**

This study will inform whether multimedia information resources, when developed using participatory design principles, are able to increase recruitment and retention of children and adolescents into trials. There is also the potential for patients to make better informed decisions through the use of multimedia information resources. The multimedia information resources also have the potential to assist with providing information on other healthcare decisions outside of clinical trials.

**Trial registration:**

ISRCTN registry: ISRCTN73136092 (doi:10.1186/ISRCTN73136092). Registered on 24 August 2016.

**Electronic supplementary material:**

The online version of this article (doi:10.1186/s13063-017-1962-z) contains supplementary material, which is available to authorized users.

## Background

The effectiveness and safety of healthcare interventions is best determined through randomised controlled trials [1, 2]. However, major barriers to the successful conduct and outcome of clinical trials are levels of recruitment and retention. In the UK, only a small proportion of trials actually recruit successfully to time and target [3–6]. Furthermore, in practice, it remains relatively uncommon for a patient to participate in a clinical trial despite the development of National Health Service organisational structures that facilitate the integration of clinical research and patient care [7], for example, the Clinical Research Networks. Inadequate recruitment or retention have implications not only for conclusive results but also external validity and generalisability of the trial findings [8].

There is now international recognition of the importance of paediatric clinical trials to inform healthcare decisions for children and adolescents [9–12]. High quality trials involving children are essential to ensure that medication and treatments used in children are effective and safe [9, 11, 12]. The lack of successful clinical trials leads to many healthcare decisions for children and adolescents being made with inadequate evidence, including evidence extrapolated from trials involving adults [12]. Only 6% of recently registered clinical trials in the UK involved children [7]. The publication rate of trials in adults has almost doubled over a 20-year period, a rate increase that is around six times higher than for paediatric trials over the same period [13]. In the past, this low rate of paediatric trials was thought to be mainly due to a concern for the vulnerability of children leading to a reluctance by clinicians to undertake clinical trials with young children [7, 14]. Nevertheless, high rates of patient or parent refusal have also been identified as a key barrier for successful completion of these trials [15], although a recent study has shown lower refusal rates for paediatric trials involving therapeutic drugs [16].

A potential barrier to recruitment and retention is the information provided to potential trial participants [2, 17, 18]. How children and parents make decisions regarding participation in research and what information is important to them remain areas of uncertainty [17, 19, 20]. Conventionally, participant information about a trial is provided in printed form. These documents should be understandable to potential trial participants and assist their decision-making [21]. However, the format of this information has received recurrent criticism, notably for being too long, difficult and technical [22–25]. Furthermore, the content of trial sheets is mostly guided by regulatory agencies and can be inconsistent with what patients want to know [17, 22, 26]. A number of studies report that trial participants do not understand information contained within participant information sheets (PISs) [27, 28] and that the information can be very wordy and overwhelming [22, 29]. Potential participants who have lower levels of literacy are most likely to be affected by this [30]. Furthermore, good graphic design, such as a structure that aids navigation of the information and visual appeal to invite and engage the reader, is often lacking in PISs. For example, written information should inform a decision about participation, but may act more as a prospectus for the trial and as a contract between researchers and the participant [31]. Re-writing, re-designing and user-testing of trial information can produce an understandable and preferred resource [32–34].

An alternative for providing information to potential trial participants is through the use of a multimedia information (MMI) resource [30, 35, 36], which presents key information using a combination of video, animation, text and audio through a website. However, research is needed to identify and evaluate different ways of presenting MMI about research to children and parents [22]. Multimedia presentation can be understood through reading, listening and watching, and allows people with different preferences to use the resource effectively [30]. MMI resources can contain all key information that would be found in a written participant information about the trial but focusing on information deemed important for children, adolescents and their parents [17, 35] when deciding whether to participate in a trial. Furthermore, MMI resources can enable the patient to select the order in which they access the information and allows people with different preferences to use the resource more effectively. Finally people’s familiarity with websites and the frequency of their use means that MMI presented on a computer (or smartphone) may now be used intuitively and easily by most people.

**Fig. 1:**
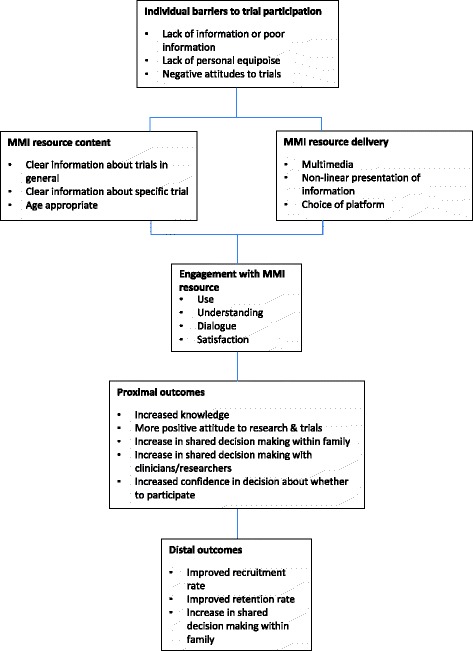
Possible pathway of effectiveness of multimedia information (MMI) resources

In educational settings, it has been estimated that individuals will remember approximately 10% of what they read, 20% of what is heard, 30% if they can visualise and hear the information, and 50% if they observe someone doing something with an explanation [37]. Multimedia, which involves using more than one medium of expression or communication, has been shown to be at least as effective as printed information [38] and often more effective in informing people [39–41]. MMI about medical procedures can improve patient knowledge [42, 43], but some have had variable impact on participant understanding [44]. However, a recent trial of children and adolescents undergoing endoscopy showed that presentation of information in electronic format produced more certain consent decisions, compared to printed information [45]. There is limited information about the effect of MMI for patients on trial recruitment rates [35, 36], although a relevant study is underway examining the use of MMI in trials recruiting adults in the UK [46, 47]. Furthermore, studies using multimedia to improve children’s understanding of clinical trials have demonstrated improved understanding when compared with those using traditional paper-based information [28]. There are a number of paediatric studies, particularly in the United States, using video for informed consent within a website format; however, the structure of this information presentation is restrictive, requiring users to view the video from start to finish [48]. MMI resources have the potential to inform and engage potential trial participants in ways that printed information can struggle to do (see Fig. 1 for possible effectiveness pathway).

The TRECA (TRials Engagement in Children and Adolescents) study will develop two MMI resources through the use of participatory design involving individual and focus group interviews with children and adolescents with long-term health conditions and their parents (Table 1) [49]. This will ensure that the MMI resources are developed to meet the needs and preferences of potential end users. The study will examine whether providing key information about clinical trials through the use of MMI resources increases recruitment and retention and enables better decision-making of children and adolescents participating in trials in the UK.

**Table 1:** Long-term health conditions

Long-term health conditions cover a wide range of conditions, that can be life-long, slowly deteriorating, potentially curable and with a variable course [49]. These conditions include (list not exhaustive):
• Diabetes
• Asthma
• Juvenile arthritis
• Cancer
• Cystic fibrosis
• Muscular dystrophy
• Early manifestation of a condition that may become chronic (e.g. acne)
Conditions which would not be included in this definition of long-term health conditions are vaccinations and those which are treated as emergency or acute conditions

## Methods/Design

### Aims

The aims of TRECA are to evaluate the potential for MMI resources to improve the quality of decision-making about participation in healthcare trials involving children and adolescents with long-term health conditions, and to assess the impact of MMI resources on trial recruitment and retention and the quality of decision-making.

The objectives of the TRECA study are:

- To involve children and adolescents with long-term health conditions, their parents and trial researchers and clinicians, in the development of two MMI resources for use when children and adolescents are being asked to consider participation in a healthcare trial.
- To obtain and analyse qualitative data from focus groups with members of key stakeholder groups (i.e. children and adolescents with long-term health conditions, parents, clinicians, trial managers) to ensure that the content and format of the MMI resources reflect their needs and preferences.
- To user-test the MMI resources with children and adolescents (and parents) to test their ability to inform potential users.
- To evaluate the MMI resources in a series of trials embedded within host trials in the UK, testing their effect on recruitment and retention rates and decision-making by comparing the effect of providing standard written participant information with provision of the MMI resource either in addition to the standard written participant information or the provision of the MMI resource alone.

### Design overview

**Fig. 2:**
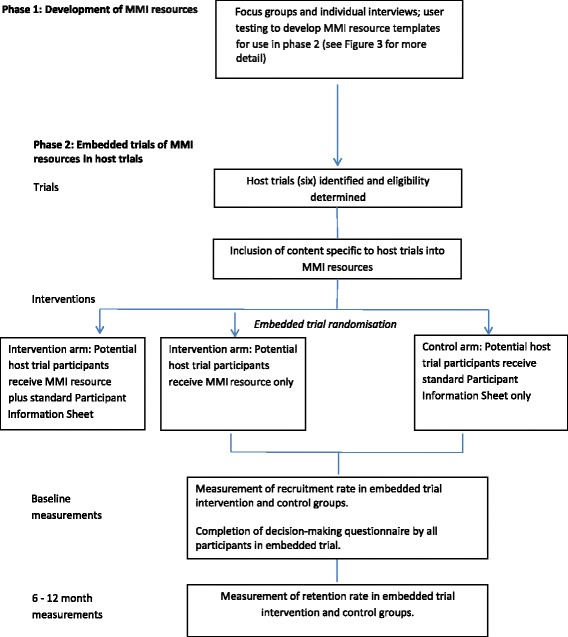
TRECA study design. *MMI* multimedia information

**Fig. 3:**
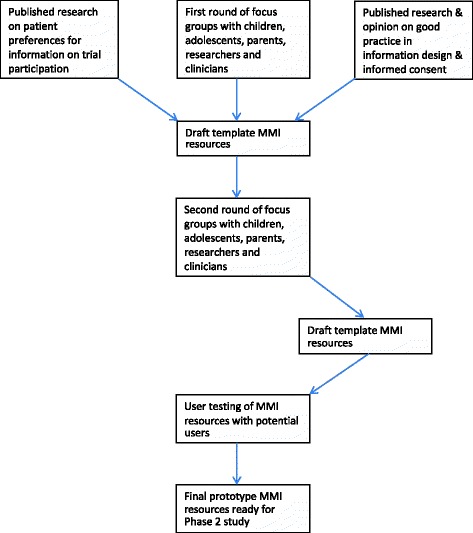
Development of the multimedia information (MMI) resources

**Fig. 4:**
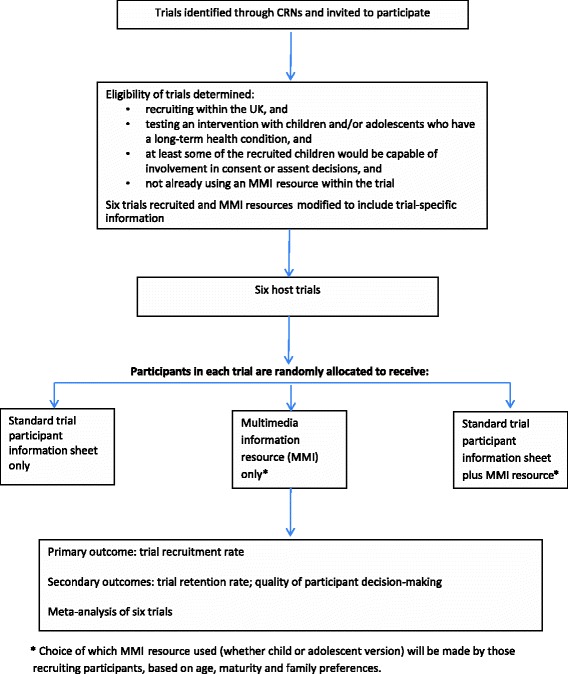
Phase two study design. *MMI* multimedia information; *CRN* Clinical Research Network

The study is divided into two phases, namely Phase one (development) and Phase two (evaluation). The study design is shown in Fig. 2. The development phase (Phase one) involves qualitative methods followed by user testing, aiming to produce two MMI resources (refer to Fig. 3 for development stages of the MMI resources), with generic elements relevant to any trial involving children and adolescents and a template for the addition of specific content for individual host healthcare trials. In the evaluation phase (Phase two), the two MMI resources will be tested in a series of embedded trials hosted within healthcare trials (refer to Fig. 4 for the Phase two study design), following the addition of a small amount of host trial-specific content to the MMI resource. The MMI resources will be tested for their impact on decisions about trial participation taken by children and adolescents and/or parents and behaviours (rates of recruitment to, and retention in, the host trials). The SPIRIT checklist describing the protocol is available as Additional file 1.

### Phase one: development

#### MMI resource development

Two MMI resources will be developed (Fig. 3) to be tested as an adjunct to, or replacement for, printed written PISs for potential child and adolescent trial participants, and their parents. The overall goal is that young potential participants and their parents will use the MMI resources to inform their decisions about entry and ongoing participation in the host trials. Both MMI resources will have generic trial information (e.g. on randomisation, study withdrawal, confidentiality, altruism and personal benefit) and a section for trial-specific information (e.g. trial purpose, intervention, number of appointments and length). The MMI resources are designed to be used by children, adolescents and their parents to assist them with making an informed decision about whether to participate in a trial.

The MMI resources will be commissioned from a specialist commercial supplier, so that their appearance and functions are professional, sophisticated and contemporary. One of the MMI resources will be intended for use by children and their parents, and the other by adolescents and their parents. Learning from a previous study looking at the development of MMI resources for adults deciding whether to participate in healthcare trials will be incorporated into the design of the current MMI resources [47, 50]. The distinct content and format of the two MMI resources will be informed by the qualitative study. The qualitative and user-testing data will also inform the topics included in each MMI resource, and the mixture of media (text, animation, video and infographics). We will also explore the potential for the MMI resources to be interactive, for example, allowing children and adolescents to post questions to the host trial research team and take mini-quizzes.

#### Participatory design: qualitative study to identify the information needs and preferences of potential users of MMI resources

Individual and focus group interviews will be undertaken with (1) children and adolescents (aged 9–11, 12–14 and 15–17 years) with long-term health conditions (Table 1); (2) parents of children and adolescents with long-term health conditions; and (3) researchers and clinicians who have experience with working in children’s clinical trials. Sampling will be purposive and will aim to achieve variation with regard to age, sex, long-term health condition, trial experience, ethnicity, and sociodemographics of participants. We plan to interview 6–10 people from each participant group, although we will increase the number as required until new data cease contributing to the analysis. For the clinician and researcher focus groups we will sample to ensure a range of roles are represented. The age split for children and adolescents is based approximately on children’s likely capacity for a role in assent/consent decisions, using an established three-part categorisation [51].

Participants will be recruited through a number of mechanisms, including Alder Hey Children’s Hospital, Generation R Young People Advisory Groups (groups with membership of children and adolescents who advise on the design of research that involves children and adolescents in the National Health Service) and three patient interest groups: PORT (Paediatric Oncology Reference Team), the Invisible Illness group and the UK Juvenile-onset Systemic Lupus Erythematosus Study Group.

Data will be collected during two rounds of individual and focus group interviews held approximately 2–3 months apart. Focus groups are preferred because they will enable discussion of ideas amongst participants, although the wishes of those who would opt for individual interviews will be accommodated. The first round will take place before the MMI resources have been designed in order to inform their content, style and delivery. The second round will take place after draft MMI resources have been produced in order to explore participants’ views on these and their suggestions for amendment.

Semi-structured individual and focus group interviews will be topic guided and focus on (1) preferences for information about research and (2) preferences for content, style and delivery of MMI resources. Participants will be prompted to discuss items of information adapted from a study [52] of adult trial participants’ information needs, and items of information identified from a study of children and adolescents’ trial experiences [53]. Items will include why the trial is being done, whether the child or adolescent has to take part or not, whether the participant will benefit personally from taking part, and who will know the child or adolescent is taking part.

Participants will also be prompted about their preferences for how the MMI resource looks and functions as informed by the website industry Webby Awards criteria [54], including general needs and preferences for MMI content, potential for interactivity (e.g. question posting, quizzes) and how the information should be presented (e.g. whether people should receive the printed information sheet before or after the MMI resource).

A pilot focus group will comprise younger children (6–8 years old) and involve showing them the developed MMI resource to explore their perspectives on whether the MMI resource is easy for them to use and understand the information provided.

Where possible, the two rounds of data collection will include the same participants to facilitate respondent validation of the ongoing analysis, with some replacement for the second round, when required. Each focus group will have between four and ten participants and be approximately 90 minutes in duration. Where participants prefer, they will be offered an individual interview either face-to-face or via Skype.

All individual and focus group interviews will be audio-recorded and transcribed verbatim.

#### Qualitative data analysis to inform the development of the MMI resources

Analysis of qualitative data will be thematic and focus on identifying what information is important for children, adolescents and their parents when deciding to participate in a trial and their thoughts on the design aspects of the MMI resource. Line by line coding will be undertaken to identify key themes within each transcript. Analysis will be led by one researcher with guidance provided by a qualitative research expert (BY) with regular meetings to discuss data interpretation. The codes will be grouped into themes and organised using NVivo version 10 software. Following the principles of participatory design, the findings on the needs and preferences of potential users will inform the development of two prototype MMI resources. We will work with patient and public involvement (PPI) members, seeking their thoughts on how to apply the findings into the development and the design of the MMI resources. The MMI resources will be informed directly based on the data obtained in the individual and focus group interviews. Where the views of the different participant groups (children/adolescents, parents and clinicians) diverge markedly, we will focus on the needs and preferences of children and adolescents.

#### User testing

Once the two MMI resource prototypes have been developed, the MMI resources will undergo user testing. We will adapt the method of user testing employed in the development of printed information, including PISs for trials [34]. This involves an iterative process with changes being made to the MMI resource in response to the data received. We anticipate having two rounds of user testing, with changes made to the MMI resources, as required, after the first round. User testing involves small participant samples to generate quantitative data, in which data patterns are interpreted to identify any problems with a piece of information that may be responsive to change. Participants will be observed using the MMI resources and then participate in brief structured interviews about the MMI resources. This will involve testing participants’ knowledge and understanding of the information in the resources and asking them to indicate where in the resource this information is located. The generated quantitative data are indicative, not definitive, and not analysed statistically. The emphasis is on identifying aspects of the information resource that might hinder understanding.

#### User testing sampling

Participants for the user testing will be recruited via a number of primary and secondary schools in Yorkshire, UK, and aim for a diverse sample of participants in terms of ethnicity, socioeconomic status and levels of academic ability, including some participants who have English as a second language. Individuals who participated in the qualitative study will not be eligible for the user testing, as user testing produces its most valid results with participants who are not already familiar with the intervention being tested [32].

We will use the conventional sampling method in user testing, namely rounds of 20 participants [55]. In the testing of the MMI resource intended for older children and adolescents, the samples of 20 will comprise a mix of parents, children and adolescents (aiming to achieve a spread of ages across the 12–17 years range). For the less complex MMI resource, rounds will comprise 10 parents and 10 younger children, aiming to achieve a spread across ages 6–11 years, with parents and children using the MMI resource together.

For each MMI resource, the two rounds of user testing will use different participants, to remove any effect of prior learning. We will ensure that the samples in the two rounds have similar profiles in terms of age and sex, to better indicate problems in the MMI resources requiring change.

#### User testing data analysis

The data derived from user testing interviews will be analysed quantitatively, although data are indicative (e.g. 80% cannot find a particular piece of information). Each item on the questionnaire will derive the following scoring criteria: finding (found, found with difficulty (i.e. found but only after a set time, usually more than 3 minutes) or not found); understanding (understood, understood with difficulty (i.e. understood but only after question rewording or repetition) or not understood).

### Phase two: evaluation of MMI resources

#### Embedded trials of MMI resources within host clinical trials – design

The effectiveness of the two MMI resources will be evaluated in a series of trials embedded within six host trials in the UK (see Fig. 4 for Phase two design) that are recruiting children and adolescents with long-term health conditions, using methods we have developed previously [47]. That is, participants in each host trial will be allocated randomly to one of two or more different intervention groups. The embedded trial (aka nested trial or ‘trial within a trial’) will be run with potential participants in each host trial – these people will be allocated randomly to receive either the MMI resource plus the printed information, the MMI resource alone, or the printed information alone, to evaluate their relative effects on recruitment rates (and the secondary outcomes).

The objective is to test the effects of the MMI resources on cognition and behaviour. The primary outcome will be whether rates of recruitment to the host trials are increased. We will also test (1) whether individuals who see the MMI resource(s) make a more informed decision about trial participation (or not); (2) whether rates of retention in the host clinical trials are increased; and (3) whether individuals are more satisfied with the process of consent or assent.

Recruitment of host trials will occur through the National Institute for Health Research Clinical Research Network, funding bodies and through investigator networks. In previous qualitative work [56], we have identified that, while trialists welcome the idea of embedding trials of recruitment interventions in their studies, these need to be compatible with the host trial design and not impose an extra workload. In the embedded trials, allocation to groups will be achieved by random number generator. Particularly, in trials in which we use individual randomisation to the MMI resources, it will be practicably very difficult to achieve concealment of randomisation. Trials will use individual or cluster randomisation. Masking of the allocation at outcome measurement will not be possible since patients cannot be masked to the information format they will receive but, as they will be unaware of the embedded information trial, a lack of masking will not bias their responses or decisions.

#### Trial eligibility criteria

Trials will be eligible for inclusion in TRECA if they are recruiting within the UK and involve testing an intervention with children and adolescents who have a long-term health condition. Within each embedded trial, participants will be children and adolescents being asked to participate in the host healthcare trial and/or their parents. This is critical, as it means that the host trial and the embedded trial have different sample sizes. For the host trial the sample comprises those children and adolescents/parents agreeing to participate; for the embedded trial of the MMI resources the sample comprises those asked to participate. In some trials, the number asked to participate is much larger, often more than double the host trial sample size.

Eligible trials should ideally have a sufficient sample size to detect a difference between groups in the embedded trial; trials will be using only printed or video participant information materials as standard (i.e. not already including an MMI resource), and will be recruiting at least some children and adolescents who have the potential to contribute to a decision about consent or assent to participation in the trial. Trials will not be included if they are only recruiting children too young to understand an MMI resource (e.g. children aged under 5 years), or only children with intellectual impairment such that understanding or use of the MMI resource is not possible.

We will aim to ensure that eligible trials vary with respect to the long-term health condition (using PRISM study criteria), the age of children and adolescents being recruited into the trial, host trials unit, the type of intervention (e.g. pharmaceutical, physical therapy, psychological), and the way that the MMI resource will be presented to patients (e.g. parent and child viewing together versus adolescent viewing separately to parent).

#### Method of embedding MMI into trials

Each of the embedded trials will use a three-arm design, in which individuals will receive the standard written trial PIS alone, the standard PIS in addition to the MMI resource, or the MMI resource alone (Fig. 2). We will consider making the written PIS available via the MMI resource, for example, by a link within the MMI resource to read or print the PIS document. This would have the advantage of being more efficient, allowing people to access both the MMI resource and the PIS via the computer. However, we will consider important practical concerns such as text readability on screens and participant preferences, and so will seek the opinions of participants in the focus groups during the development study phase. We will also measure the number of page views and the frequency which individual elements of the MMI resource are viewed.

Patients allocated to the control arm of the embedded trial will be given the printed PIS only (as is usual). Those allocated to one of the intervention arms will receive either the MMI resource alone or the printed PIS and the MMI resource(s). We will not determine the order in which participants access the PIS and MMI resource (for those participants who receive both) and will leave this for the host trial to determine, to suit the practical demands of patient recruitment. However, we will ask the host trial to record the order in which participants are given and access the PIS/MMI resource, and report this observation in the report of each embedded trial. The MMI resources will be presented in the clinic on a computer or dedicated tablet computer. Participants will also be able to access the MMI resources at home (via smartphone or a tablet or PC) via a link that is emailed to them. In some circumstances, home viewing will take place before the patient’s decision on clinical trial participation has been taken. Some patients will also want to be given access to the MMI resource after they have decided to take part in the host healthcare trial, just as they would if they had been given standard printed information only.

#### Outcome measures

The primary outcome will be the rates of recruitment to each host trial. We will calculate the proportion of patients who agree to participate from the total number approached, for each arm of the embedded trial. This study is investigating whether MMI resources improve the quality of decision-making, related to individuals being more informed about the trial. Improved decision-making may have no impact on trial recruitment rates, although it is also possible that it could either increase or decrease rates as a result of individuals being more informed. It is therefore important that we also measure secondary outcomes of retention in the trials, and quality of decision-making. We will measure retention by obtaining data on the number and timing of drop outs from each host trial. The quality of decision-making by potential host trial participants will be measured through the completion by children, adolescents and parents (as relevant) of a decisional scale, adapted from one used within the REFORM trial [57] and drawing conceptually on the SURE [58] and DelibeRATE scales [59, 60]. We will report study results in line with published guidelines [61].

#### Sampling considerations

The projected effect of the MMI resources will vary in size according to the setting of the host trial, the background recruitment rate, and the intervention being tested in the host trial. This makes it difficult to establish a sample size calculation for each of the six embedded trials. The effectiveness of the MMI resources is being assessed against three outcome measures, namely host trial recruitment rate, quality of decision-making on participation (or not) and host trial retention rate. Results from each embedded trial will be combined in a prospective meta-analysis for decision scores and recruitment rate data from all six host trials participating in the TRECA study.

### PPI

The Investigators and research team have a strong commitment to PPI in the TRECA Study. TRECA has a Patient and Parent Advisory Group which will play a key role in reviewing and providing input into documentation used in the various stages of the study, including topic guides and prototype MMI resources. The Patient and Parent Advisory Group will also participate in the piloting of user testing questionnaires to ensure that the question wording and length are appropriate. Two members of the Patient and Parent Advisory Group are also members of the TRECA Study Advisory Group and one or two PPI members will be involved in co-facilitation of the second set of focus groups.

## Discussion

Whilst trials have been undertaken for many decades in medicine, limited data is available about the decision-making processes for trial participants and which information is important for them when considering whether to participate [2, 21, 52]. Studies that have examined the information needed for potential participants to make such decisions often do not include the level of detail that participants wanted to receive [52]. The numbers of studies looking at this issue in relation to children, adolescents and their parents, is limited [19, 20, 22, 53].

The TRECA study will identify the information that children and adolescents consider to be most important when deciding whether or not to participate in clinical trials. Informed by the principles of participatory design, we will produce MMI resources that aim to meet children, adolescents and their parents’ needs and preferences for information content and presentation. In particular, this should lead to improved patient information resources that offer relevant information in a way that is accessible to all potential participants, whilst also increasing their understanding of clinical trials in general. Ultimately, it is anticipated that improving participant information resources will increase participation in clinical trials. Specifically, this study will lead to the development of two MMI resources that are suitable for children and adolescents invited to future clinical trials. The findings may also be of use to researchers in different settings, such as education or mental health, in order to better inform and recruit participants to studies. It may also identify principles that are transferable to other medical settings where providing accessible information is crucial, for example, regarding patient decisions about hospital procedures or treatment options.

Results from the TRECA study will be published in peer-reviewed journals and disseminated widely through conferences and events. Where possible, we will also use social media to publicise the findings. In particular, we will aim to engage with health charities, self-help groups and lobbyists, with a view to informing children and adolescents with a variety of long-term health conditions who may be involved in research.

### Trial status

Phase one of the study has commenced. Recruitment for Phase two has not yet commenced.

## Additional file

Additional file 1: Standard Protocol Items Recommendations for Interventional Trials (SPIRIT) 2013 checklist. (DOC 122 kb)

## Abbreviations

MMI: multimedia information
PIS: participant information sheet
PPI: patient and public involvement
TRECA: TRials Engagement in Children and Adolescents
